# Supplementary material for: Phage Intolerance Impacts Antibiotic Susceptibility and Virulence in Staphylococcus aureus
Source: Curr Microbiol. 2026 Jun 24;83(8):452. doi: 10.1007/s00284-026-05034-6 (PMC13294244; doi:10.1007/s00284-026-05034-6)
Supplement: Supplementary file 1 — Supplementary Material 1 [file 284_2026_5034_MOESM1_ESM.pdf]

Table S1. Bacterial strains used in this study

Bacteria	Strain no.	Strain description	Source
S. xylosus	HI5917	DD-34, propagative host for Stab21	(1)
S. aureus	HI5882	USA300 JE2	(2)
	HI5830	USA300 JE2 Δ*tagO*	(3)
	HI2157	RN4220, phage cured lab strain	(4)
	HI2156	8325-4, 8325 with prophages ϕ11, ϕ12, and ϕ13 removed, propagative host for ϕIPLA-RODI and K	(5)
	HI5911	K4 (RH7), phage tolerant JE2 mutant	This study
	HI5912	R4 (RH9), phage tolerant JE2 mutant	This study
	HI5881	K1 (RH10), phage tolerant JE2 mutant	This study
	HI5913	S4 (RH11), phage tolerant JE2 mutant	This study
	HI5882	R1 (RH12), phage tolerant JE2 mutant	This study
	HI5883	R2 (RH18), phage tolerant JE2 mutant	This study
	HI5914	K5 (RH19), phage tolerant JE2 mutant	This study
	HI5884	S1 (RH20), phage tolerant JE2 mutant	This study
	HI5915	R5 (RH21), phage tolerant JE2 mutant	This study
	HI5885	K2 (RH22), phage tolerant JE2 mutant	This study
	HI5886	S2 (RH23), phage tolerant JE2 mutant	This study
	HI5887	S3 (RH30), phage tolerant JE2 mutant	This study
	HI5888	K3 (RH31), phage tolerant JE2 mutant	This study
	HI5889	R3 (RH32), phage tolerant JE2 mutant	This study
	HI5614	JE2 pLOW-dcas9_aad9	(6)
	HI5620	JE2 pLOW-dcas9_aad9, pVL2336-no target	This study
	HI5916	JE2 pLOW-dcas9_aad9, pVL2336-tagO	This study
	HI5890	JE2 pLOW-dcas9_aad9, pVL2336-cshA	This study
	HI5895	JE2 pLOW-dcas9_aad9, pVL2336-ligA	This study
	HI5891	JE2 pLOW-dcas9_aad9, pVL2336-deoC1	This study
	HI5896	JE2 pLOW-dcas9_aad9, pVL2336-femA	This study
	HI5894	JE2 pLOW-dcas9_aad9, pVL2336-potAB	This study

	HI5892	JE2 pLOW-dcas9_aad9, pVL2336-merR	This study
	FM148	JE2 pSK9067	This study
	FM149	K1 pSK9067	This study
	FM150	R1 pSK9067	This study
	FM151	R3 pSK9067	This study
	FM143	JE2 pSK9067-deoC1	This study
	FM144	K1 pSK9067-deoC1	This study
	FM145	R1 pSK9067-deoC1	This study
	FM146	R3 pSK9067-deoC1	This study
E. coli	HI4273	IM08B	(7)
	HI5640	IM08B pLOW-dcas9_aad9	(8)
	HI5641	IM08B pVL2336-tagO	(9)
	HI5875	IM08B pVL2336-cshA	This study
	HI5878	IM08B pVL2336-ligA	This study
	HI5873	IM08B pVL2336-deoC1	This study
	HI5874	IM08B pVL2336-femA	This study
	HI5876	IM08B pVL2336-potAB	This study
	HI5877	IM08B pVL2336-merR	This study
	FM133	IM08B pSK9067-deoC1	This study

**Plasmids**	**Name**	**Description**	**Source**
	pLOW	Constitutively expressed CRISPRi vector	(8)
	pVL2336	IPTG inducible expression vector	(9)
	pSK9067	IPTG inducible shuttle vector	(10)

Table S2. Lytic bacteriophages used in this study.

Bacteriophages	Propagative strain	Source
ϕIPLA-RODI	*S. aureus* 8325-4	*(11)*
K	*S. aureus* 8325-4	*(12)*
Stab21	*S. xylosus* DD-34	*(1)*

Table S3. Antibody-related reagents and resources.

Name	SOURCE	IDENTIFIER
Clone 4461: anti-α-1,4-GlcNAc-WTA Fab	Nina van Sorge Amsterdam UMC, NL (12)	N/A
Clone 4462: anti-β-1,4-GlcNAc-WTA Fab	Nina van Sorge Amsterdam UMC, NL (12)	N/A
Goat F(ab')2 anti-human-kappa-AF647, secondary antibody	SouthernBiotech	2062-31
BSA (bovine serum albumin)	Sigma-Aldrich	Cat#A7030

Table S4. Gene putative function from COG/PFAM associations.

Gene number	Gene name	COG	Description	Putative Function
B7H15_00770	*deoC1*	COG0274	Nucleotide transport and metabolism	Metabolism
B7H15_05705	*potA*	COG3842	Amino acid transport and metabolism	Metabolism
B7H15_05710	*potB*	COG1176	Amino acid transport and metabolism	Metabolism
B7H15_11560	*cshA*	COG0513	Replication, recombination and repair	Replication
B7H15_07240	*femA*	pfam02388*	femAB family, peptidoglycan sysnthesis	Cell wall
B7H15_10620	*ligA*	COG0272	Replication, recombination and repair	Replication
B7H15_13250	*merR*	pfam13411*	transcriptional regulator	Regulation
B7H15_04185	*tagO*	COG0472	Cell wall/membrane/envelope biogenesis	Cell wall

**no COG assigned, pfam used instead*

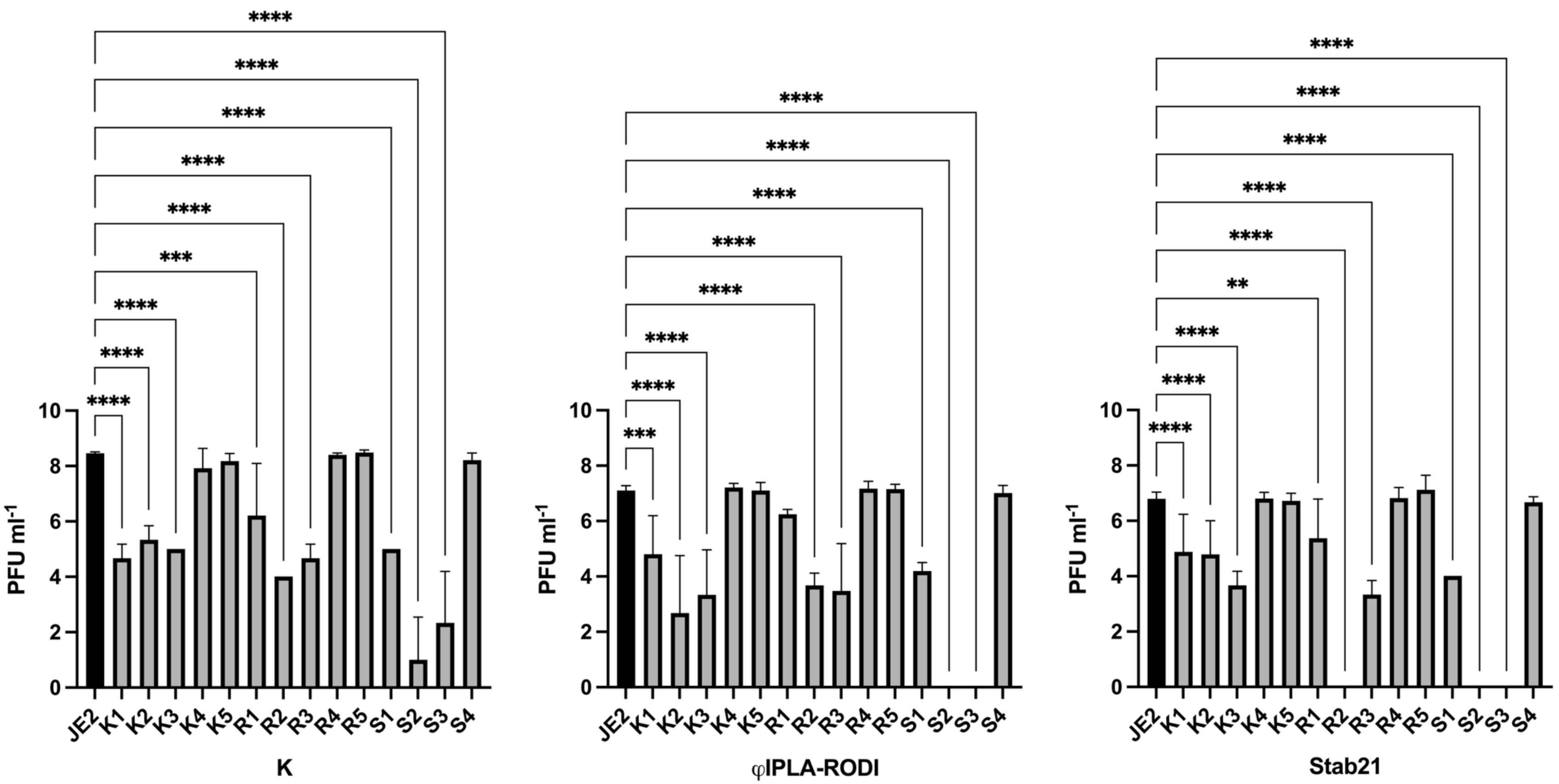

Figure S1. Phage titer screen of potential phage resistant clones. A. PFU ml^{-1} of phage K **B.** ϕIPLA-RODI, and **C.** Stab21 for suspected phage resistance colonies compared to the parental JE2. Data show mean with standard deviation for 6 biological replicates that was log transformed. Ordinary one-way ANOVAs with Dunnett's multiple comparisons test were performed comparing clones to the parental JE2. Significant differences are indicated by one (p <0.05), two (p <0.01), three (p <0.001) or four (p <0.0001) asterisks (*). Clone names indicate which of the phages was used in the resistance development protocol (K for phage K, R for ϕIPLA-RODI, S for Stab21).

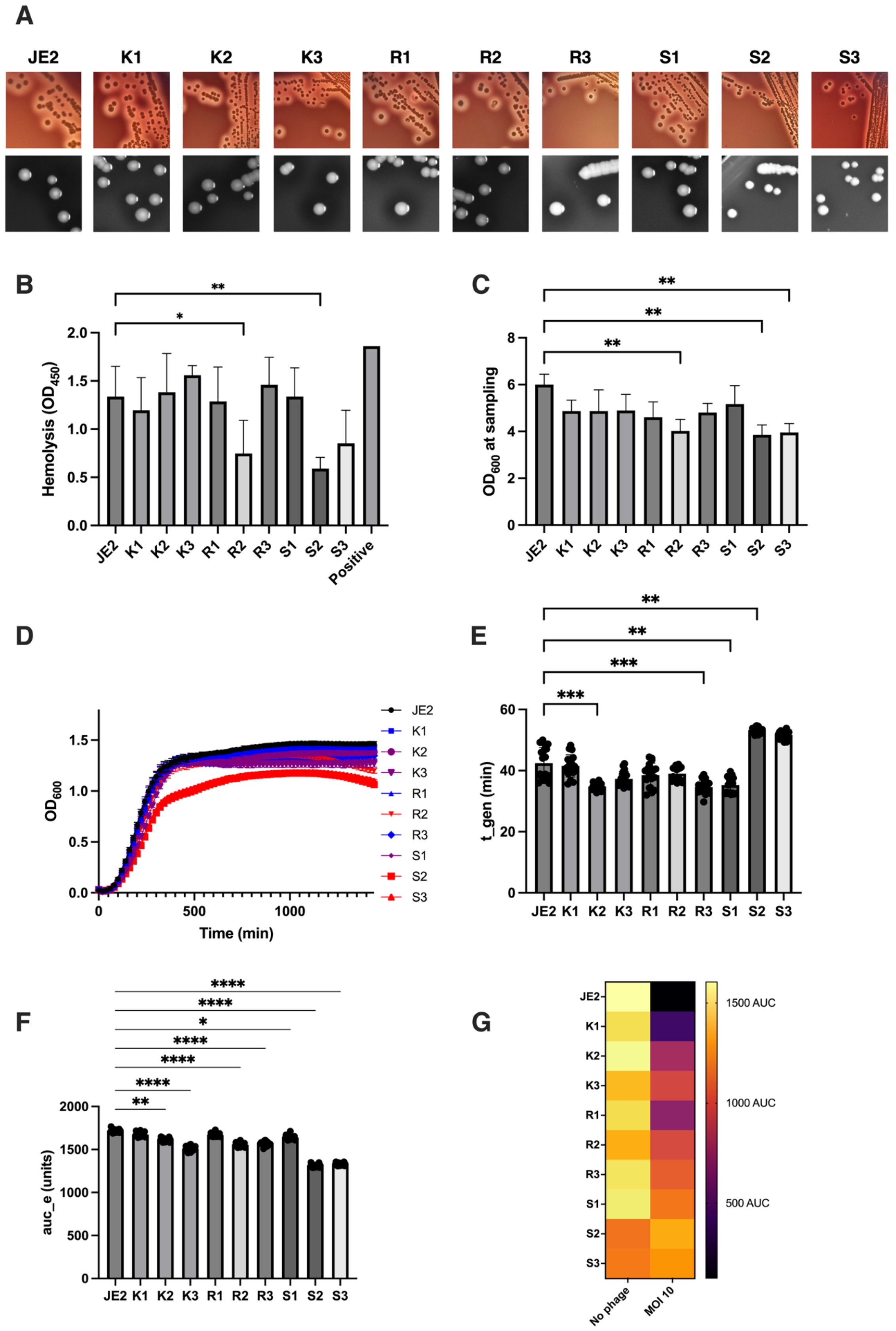
A
JE2
K1
K2
K3
R1
R2
R3
S1
S2
S3
B
Hemolysis (OD_{450})
Positive
C
OD_{600} at sampling
D
OD_{600}
Time (min)
E
t_gen (min)
F
auc_e (units)
G
1500 AUC
1000 AUC
500 AUC
No phage
MOI 10

Figure S2. Hemolysis quantification and growth phenotypes for the phage-resistant clones. A. Colony morphology and hemolysis. **B.** Quantitative hemolysis assay for the parental JE2 and phage resistant clones. **C.** OD_{600} of the bacterial sample used for the hemolysis assay at time of sampling. **D.** Growth curves for JE2 parental strain and phage resistant clones**. E.** Generation time (t_gen). **F.** Area under the curve (empirical). For B, C, E and F, ordinary one-way ANOVAs were performed, comparing each clone to the parental JE2, with Dunnett's multiple comparisons test. Significant differences are indicated by one (p <0.05), two (p <0.01), three (p <0.001) or four (p <0.0001) asterisks (*). Data shows 3 biological replicates, with 6 technical replicates. **G.** Heatmap of Area Under the Curve (empirical) for collection of mutants grown with or without addition of phage K to MOI 10 with unit legend shown on right. Data shows 2 biological replicates.

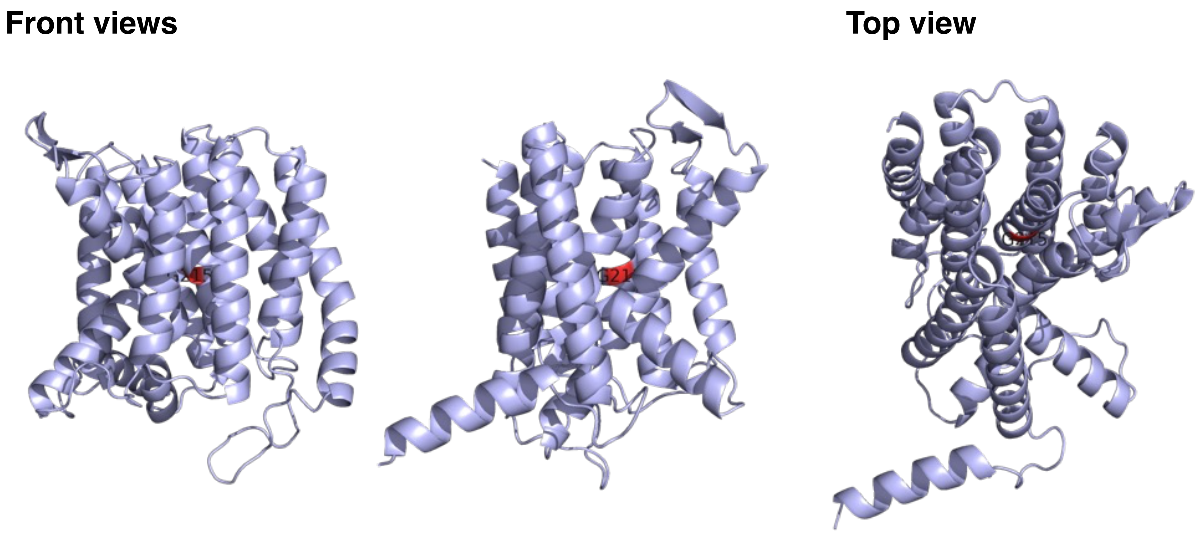

Figure S3. '**Front views'**, two different faces of the predicted TagO structure, '**Top view**' of predicted TagO structure. G215 is labelled and indicated in red, this was mutated from glycine to arginine in clone S2 (G215R) changing the side chain from nonpolar and small, to a large, positively charged polar side chain.

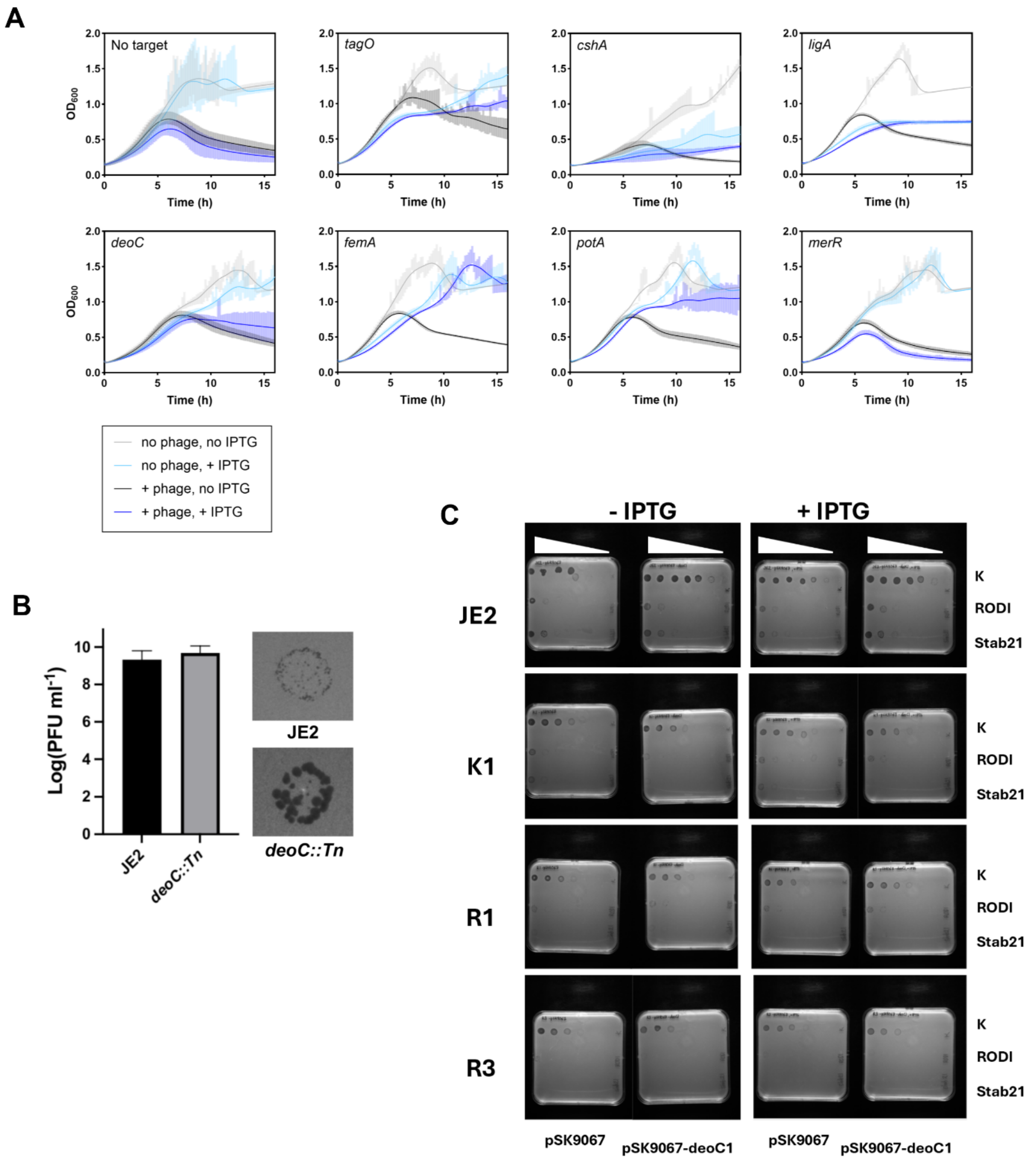

Figure S4, Validation of genes linked to increased phage tolerance. A. Graphs show growth curves for *S. aureus* strains with (light/dark blue, 250 μM) or without (grey/black, 0 μM) IPTG-induced CRISPRi knockdown of genes with SNPs in the phage tolerant clones. Each graph represents the knockdown targeting one gene, as indicated by the gene name in the top-left corner. As in the legend, graphs plot 4 conditions:

exposure to phage buffer only (no phage, no IPTG; grey), with IPTG (no phage, +IPTG: sky blue), with phage K but without IPTG (phage, no IPTG: black), and with phage K and IPTG (phage, +IPTG: dark blue). Phage K concentration was MOI 10^{-4}, except for *tagO* and *deoC* where it was MOI^{-5}. Graphs show the mean of 6 biological replicates, with shading indicating standard error of mean. The curves representing the mean were smoothed using LOWESS (10 points in smoothing window). **B, Left**, Log transformed PFU ml^{-1} for JE2 wt and JE2 *deoC1::Tn*. Data shows 4 biological replicates. Welch's t test showed no significant differences. **Right**, phage plaque phenotypes. **C.** JE2 wt and *deoC1* mutated clones K1, R1 and R3 complemented with wildtype *deoC1* allele exposed to phage pressure with induction of promoter Pspac (+/- IPTG). Data shows 1 biological replicate.

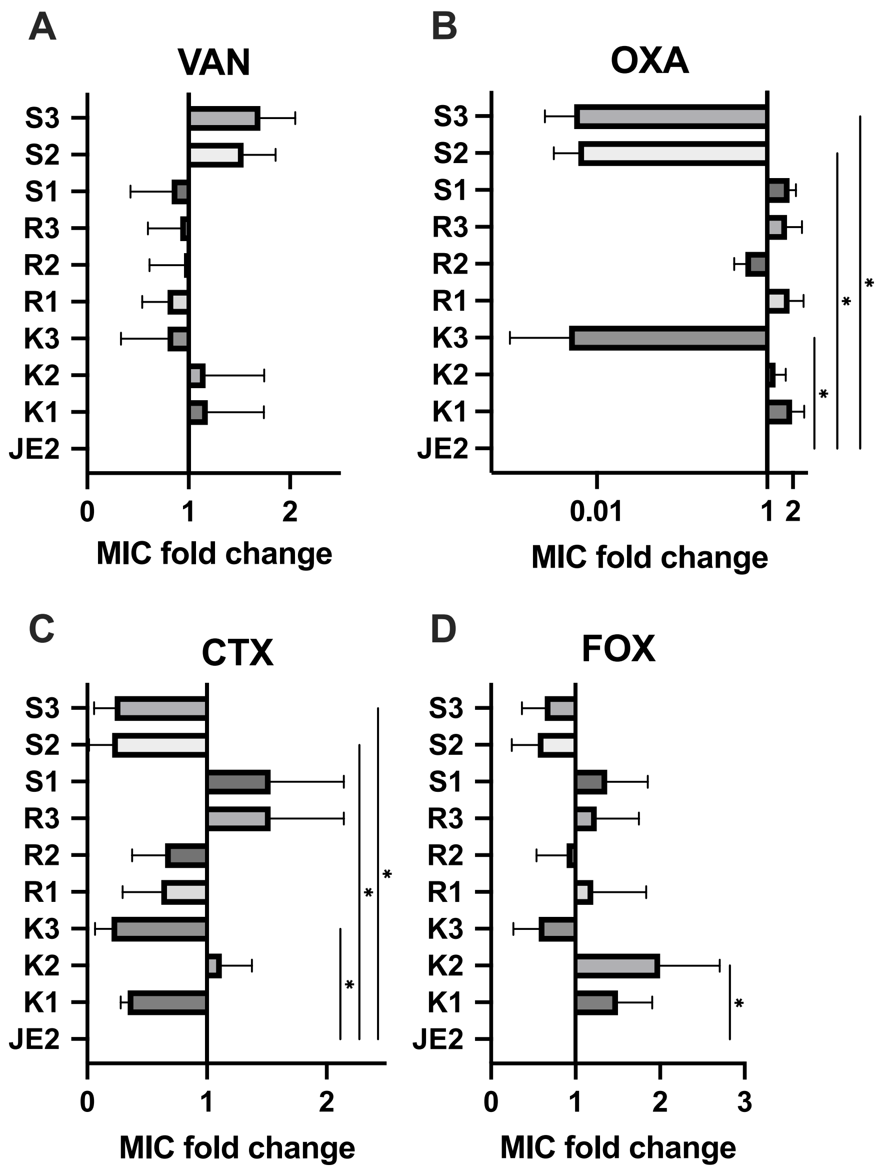

Figure S5. Fold change of Etest MIC measurements. Fold change calculations for Figure 3 MIC data, comparing phage resistant clones to parental JE2 for **A** vancomycin (**VAN)**, **B** oxacillin (**OXA**), **C** cefotaxime (**CTX**), and **D** cefoxitin (**FOX**). Ordinary one-way ANOVAs with Dunnett's multiple comparisons test were performed. Significant differences are indicated by one (*p* <0.05), two (*p* <0.01), three (*p* <0.001) or four (*p* <0.0001) asterisks (*).

References

1. Oduor JMO, Kiljunen S, Kadija E, Mureithi MW, Nyachieo A, Skurnik M. Genomic characterization of four novel Staphylococcus myoviruses. Archives of Virology. 2019;164(8):2171-3.
2. Kennedy AD, Otto M, Braughton KR, Whitney AR, Chen L, Mathema B, et al. Epidemic community-associated methicillin-resistant Staphylococcus aureus: Recent clonal expansion and diversification. Proceedings of the National Academy of Sciences. 2008;105(4):1327-32.
3. Slavetinsky J, Lehmann E, Slavetinsky C, Gritsch L, van Dalen R, Kretschmer D, et al. Wall Teichoic Acid Mediates Staphylococcus aureus Binding to Endothelial Cells via the Scavenger Receptor LOX-1. ACS Infectious Diseases. 2023;9(11):2133-40.
4. Kreiswirth BN, Löfdahl S, Betley MJ, O'Reilly M, Schlievert PM, Bergdoll MS, et al. The toxic shock syndrome exotoxin structural gene is not detectably transmitted by a prophage. Nature. 1983;305(5936):709-12.
5. Novick R. Properties of a Cryptic High-Frequency Transducing Phage in Staphylococcus aureus. VIROLOQY. 1967;33(1):155-66.
6. Barbuti MD, Lambert E, Myrbraten IS, Ducret A, Stamsas GA, Wilhelm L, et al. The function of CozE proteins is linked to lipoteichoic acid biosynthesis in Staphylococcus aureus. mBio. 2024;15(6):e0115724.
7. Monk IR, Tree JJ, Howden BP, Stinear TP, Foster TJ. Complete Bypass of Restriction Systems for Major Staphylococcus aureus Lineages. mBio. 2015;6(3):e00308-15.
8. Myrbraten IS, Stamsas GA, Chan H, Morales Angeles D, Knutsen TM, Salehian Z, et al. SmdA is a Novel Cell Morphology Determinant in Staphylococcus aureus. mBio. 2022;13(2):e0340421.
9. Liu X, de Bakker V, Heggenhougen MV, Marli MT, Froynes AH, Salehian Z, et al. Genome-wide CRISPRi screens for high-throughput fitness quantification and identification of determinants for dalbavancin susceptibility in Staphylococcus aureus. mSystems. 2024;9(7):e0128923.
10. Brzoska AJ, Firth N. Two-plasmid vector system for independently controlled expression of green and red fluorescent fusion proteins in Staphylococcus aureus. Appl Environ Microbiol. 2013, 79(9):3133-6.
11. Gutiérrez D, Vandenheuvel D, Martínez B, Rodríguez A, Lavigne R, García P. Two Phages, phiIPLA-RODI and phiIPLA-C1C, Lyse Mono- and Dual-Species Staphylococcal Biofilms. Applied and Environmental Microbiology. 2015;81(10):3336-48.
12. O'Flaherty S, Ross RP, Meaney W, Fitzgerald GF, Elbreki MF, Coffey A. Potential of the Polyvalent Anti-Staphylococcus Bacteriophage K for Control of Antibiotic-Resistant Staphylococci from Hospitals. Applied and Environmental Microbiology. 2005;71(4):1836-42.
